# Evidence of Paternal Effects on Telomere Length Increases in Early Life

**DOI:** 10.3389/fgene.2022.880455

**Published:** 2022-05-17

**Authors:** Sophie Bennett, Antje Girndt, Alfredo Sánchez-Tójar, Terry Burke, Mirre Simons, Julia Schroeder

**Affiliations:** ^1^ Division of Biology, Imperial College London, London, United Kingdom; ^2^ UK Centre for Ecology & Hydrology, Wallingford, United Kingdom; ^3^ Department of Evolutionary Biology, Max Planck Institute for Ornithology, Seewiesen, Germany; ^4^ Department of Evolutionary Biology, Bielefeld University, Bielefeld, Germany; ^5^ School of Biosciences, Ecology and Evolutionary Biology, University of Sheffield, Sheffield, United Kingdom

**Keywords:** telomere dynamics, intergenerational effect, z-linked inheritance, transgenerational effects, Lansing effect, *Passer domesticus*

## Abstract

Offspring of older parents in many species have decreased longevity, a faster ageing rate and lower fecundity than offspring born to younger parents. Biomarkers of ageing, such as telomeres, that tend to shorten as individuals age, may provide insight into the mechanisms of such parental age effects. Parental age may be associated with offspring telomere length either directly through inheritance of shortened telomeres or indirectly, for example, through changes in parental care in older parents affecting offspring telomere length. Across the literature there is considerable variation in estimates of the heritability of telomere length, and in the direction and extent of parental age effects on telomere length. To address this, we experimentally tested how parental age is associated with the early-life telomere dynamics of chicks at two time points in a captive population of house sparrows *Passer domesticus*. We experimentally separated parental age from sex effects, and removed effects of age-assortative mating, by allowing the parent birds to only mate with young, or old partners. The effect of parental age was dependent on the sex of the parent and the chicks, and was found in the father-daughter relationship only; older fathers produced daughters with longer telomere lengths post-fledging. Overall we found that chick telomere length increased between the age of 0.5 and 3 months at the population and individual level. This finding is unusual in birds with such increases more commonly associated with non-avian taxa. Our results suggest parental age effects on telomere length are sex-specific either through indirect or direct inheritance. The study of similar patterns in different species and taxa will help us further understand variation in telomere length and its evolution.

## Introduction

Parent age at conception is often associated with their offspring’s life-history, with offspring of older parents commonly having reduced reproductive success and longevity (Priest et al., 2021a; Schroeder et al., 2015; Heidinger et al., 2016; Monaghan et al., 2020), but see (Travers et al., 2021). Moreover, in some species, offspring of older parents experience higher rates of senescence, cellular ageing, and decreased longevity that may be associated with telomere attrition compared to their older siblings (Bouwhuis et al., 2010; Torres et al., 2011; Broer et al., 2013). While some studies do not find such cross-generational effects of age (Unryn et al., 2005; Froy et al., 2017), the cross-generational effects of age are reported across a wide range of taxa from rotifers (King, 1983) and insects (Priest et al., 2021a) to birds and mammals (Haussmann et al., 2003; Bize et al., 2009), termed the Lansing effect (Lansing, 1947).

One biomarker associated with biological age and longevity is the relative length of telomeres, the chromosome capping structures consisting of TTAGGG base pair repeats in vertebrates (Mather et al., 2011; Heidinger et al., 2012; Vedder et al., 2021). At each cell division telomeres shorten as the very ends of chromosomes are not replicated, known as the end-replication problem (Levy et al., 1992). Telomeres also partly function to prevent reactive oxygen species from damaging coding DNA and are damaged themselves in the process (Aubert and Lansdorp, 2008). The activity levels and expression of telomerase, the enzyme capable of elongating telomeres, decline rapidly in early life and are tissue specific (Taylor and Delany, 2000). Together these processes lead to a gradual telomere shortening over an individual’s lifetime (Finkel and Holbrook, 2000; Aubert and Lansdorp, 2008), which is why telomere length has been investigated as a biomarker for biological age (Zglinicki and Martin-Ruiz, 2005; Mather et al., 2011). However, whether there is a direct causal link between telomere length and ageing remains unclear (Boonekamp et al., 2013; Simons, 2015).

In birds, telomere loss is fastest in early life and an initially longer telomere length is associated with longer subsequent lifespans in captive and wild bird populations (Salomons et al., 2009; Heidinger et al., 2016; Wilbourn et al., 2018). There is evidence for telomere length being heritable in birds (Vedder et al., 2021), and telomere dynamics have been associated with sex-specific parental age and telomere length (Asghar et al., 2015; Reichert et al., 2015). However, the direction of the association between telomere length, and maternal and paternal age varies even within bird species (Dugdale and Richardson, 2018; Heidinger and Young, 2020). In some bird species, the offspring of older parents may have shorter telomeres and a faster attrition rate, especially in early development (Heidinger et al., 2016). Other studies find this effect only in relation to older mothers (Asghar et al., 2015), or fathers (Horn et al., 2011; Noguera et al., 2018; Sparks et al., 2021). Another body of studies find a positive relationship between parental age and early life telomere lengths in offspring (fathers: Heidinger et al., 2021; mothers: Sparks et al., 2021). Consequently there is a great need for additional studies investigating the complexities of the relationship between parental ages and offspring telomere lengths.

Between taxa, studies on the heritability of telomere length are conflicting. The heritability of telomere length can be sex-specific and is often larger in the heterogametic sex; suggesting some degree of maternal inheritance in birds (Horn et al., 2011; Asghar et al., 2015; Reichert et al., 2015; Marasco et al., 2019) and paternal inheritance in humans (Njajou et al., 2007; Nordfjäll et al., 2009; Eisenberg et al., 2017). However, homogametic inheritance of telomere length has been identified in humans (Broer et al., 2013), in birds (Bouwhuis et al., 2018; Bauch et al., 2019), and in lizards (Olsson et al., 2011). Furthermore, a lack of heritability has also been found in several bird species (Heidinger et al., 2012; Atema et al., 2015; Kucera, 2018). Overall then, parental age effects on offspring telomere length, dynamics and heritability are complex, and vary in extent and direction within and between taxa.

Here, we test for sex-specific, age-related parental effects on chick telomere length dynamics in captive house sparrows *Passer domesticus*. By pairing different age categories of parents, we experimentally test the hypothesis that chicks of older parents have shorter telomeres and faster telomere attrition than chicks from younger parents.

## Materials and Methods

### Study Species and Experimental Design

We used captive house sparrows at the Max Planck Institute for Ornithology, Seewiesen, Germany, during the breeding season of 2014 (May-July). We used 42 pairs of male and female sparrows, which were assigned to four treatments, each with an equal sex ratio and a uniform distribution of ages across both sexes to control for age-assortative mating. We experimentally bred pairs in one of four age combinations: old-female/old-male (OO, *n* = 8 pairs included in this study), old-female/young-male (OY, *n* = 11 pairs), young-female/old-male (YO, *n* = 13 pairs), and young-female/young-male (YY, *n* = 10 pairs). Young birds hatched the preceding summer. Old (O) sparrows were age 4 years and older, although most individuals were 7 years or older (Males: 8 years = 2, 9 years = 21; Females: 4 years = 1, 7 years = 10, 8 years = 4, and 9 years = 1). The difference in age distribution between females and males corresponded to that observed in the wild, where females have a shorter lifespan than males (Schroeder et al., 2012). We did not use birds of an intermediate age because in wild house sparrows, reproductive senescence may start at 3 years of age for females (Schroeder et al., 2012), or 5 years in males (Hsu et al., 2017). Each treatment group was split in two separate breeding groups located in separate semi-outdoors aviaries. Aviaries had a dimension of 1.2 m × 4.0 m × 2.2 m (length × width × height). Each aviary contained between 24–31 individuals. As the outside of aviaries was a semi-permeable mesh the birds experienced essentially natural environmental conditions. Aviaries also received some additional artificial lighting around dawn and dusk to compensate for slightly reduced light levels inside the aviaries at this time of day compared to the local natural conditions. Each aviary contained 15.3 (s.d = 4.9) males and 14.6 (s.d = 2.4) females of the respective age class. Bird husbandry is described in more detail in Girndt et al. (2017).

Each aviary was equipped with one more nest box than breeding pairs to reduce male-male competition for nest boxes. Sparrows were then allowed to naturally display, form pair bonds, choose a mate restricted by the age class present, and raise their young (Girndt et al., 2018). We systematically monitored breeding and identified the parents attending each nest box by observing the individual birds’ colour ring combinations.

### Blood Sample Collection

We took blood samples from all chicks before they fledged, 0.5 months after they hatched (samples taken at 12 days post-hatching, *n* = 75). Blood samples were collected from the brachial vein of chicks using 1 mm capillary tubes and stored in 1 ml of 96% ethanol. After fledging, chicks remained in the same aviary as their parents and siblings, and were blood sampled again 2.5–3 months later (*n* = 59, samples taken at 83–115 days post-hatching). We obtained second samples for an additional 15 chicks however, these samples were taken at significantly different time points (24–74 days of age) due to logistical constraints and so were unsuitable for inclusion in this study. We collected samples of 56 individuals at both time points to test for within-individual changes. After collection blood samples were stored at room temperature in ethanol until DNA extraction.

### DNA Extraction and Quantification

DNA for all 0.5 months samples and a minority of 3 months samples were extracted just prior to qPCR processing. However, the majority of 0.5 months samples had DNA extracted concurrent with their sampling (up to 18 months previously) and were then frozen at −20°C until being thawed for this study. Following standard DNA extraction (Richardson et al., 2001), the DNA concentration of all samples was measured using a ThermoScientific NanoDrop8000 Spectrophotometer and sample concentration was to 20–30 ng/ml to ensure similar amplification of samples during qPCR. Where necessary, samples were diluted with T10E0.1 (10 mM Tris-HCl, pH 8.0, 0.1 mM EDTA, pH 8.0) or concentrated using a ThermoScientific Savant DNA SpeedVac Concentrator. Purity of samples was checked through measurement of 260/280 absorbance ratios; ratios were between 1.7–2.0 for all samples.

### Estimation of Telomere Length

We used multiplex qPCR to determine relative telomere length. We determined “T” as the number of telomere repeats and “S” as the number of control gene repeats using GAPDH as a reference gene. We then used the T/S ratio as a proxy for telomere length. The four DNA primers we used are described in Criscuolo et al. (2009). We used DNA from house sparrows not included in this analysis as a golden sample dilution standard at five DNA concentrations of approximately 80, 20, 5, 1.25, and 0.31 ng/ml, on each plate. We then used these standards to produce a standard curve for all analysed samples. In each well we added 1.5 μl of DNA sample, 0.9 μl of each primer, 10 μl of Sybr®Select Master Mix and 4.9 μl ddH_2_O. We ran each plate with an equal number of 0.5 and 3 months sample pairs from the same individual to account for any potential sample and plate effects when comparing within-individual changes in telomere length. We ran 42 samples, the five standards and a negative (with all components except a DNA sample) in duplicate on each 96-well plate. We ran the qPCR cycling conditions using QuantStudio 12kFlex Software v1.2.2 following the cycle timings given in Cawthon (2009). We analysed the software output to calculate the T/S ratio in each sample using a custom script that performed background subtraction, thresholding and standard curve correction (code provided). We altered the thresholds for the standard curve of the telomere and GAPDH primers for each plate based on amplification plots resulting in efficiencies of between 99.3–99.7 for GAPDH and 99.3–105.8 for telc and telg. The standard curve for each plate had an R^2^ of 0.99 and the intra- and inter-plate variation coefficients all met adequate levels (Cawthon, 2009). We also ran a melt curve to confirm whether the expected two products were generated in the reaction. Additionally, we checked all plate amplification curves to see if DNA was present in the control, as this would indicate contamination. In all plates DNA was absent, apart from very late amplification due to primer dimerization. We repeated any sample duplicates that had a standard deviation of >0.05 following thresholding and used the mean T/S ratio of duplicates in our analysis. To test the reliability of these measurements we also calculated the repeatability of the T/S ratios at both time points using the individually duplicated T/S measurements, using the R package ‘rptR’ v.0.9.22 (Stoffel et al., 2017) with 1000 bootstrap iterations. Based on duplicates of the same sample the sample repeatability of the T/S ratios at 0.5 months was 0.98 (95% confidence interval: 0.97, 0.99), and at 3 months it was 0.99 (95% confidence interval: 0.99, 0.99). The individual repeatability of T/S ratios between the two time points was 0.27 (95% confidence interval: 0.01, 0.51), a similar value to the early life telomere length repeatability estimated by Heidinger et al. (2021) (r = 0.28, range 0.18–0.5). All samples were analysed for telomere length at the same time and had a similar shelf time (Lieshout et al., 2020). All reagents and equipment were produced by Thermo Fisher Scientific, Waltham, Massachusetts, US.

### Ethical Note

The Government of Upper Bavaria, Germany, approved the care, handling and husbandry of all birds in this study and granted a license for animal experiments to JS (Nr311.5–5682.1/1-2014-024).

### Statistical Analysis

In our analyses, T/S ratios of chicks at 0.5 months old are referred to as T/S_0.5_ and samples at 2.5–3 months old as T/S_3_. We tested for a change in telomere length over the 2.5 months period by running a linear mixed effects model (LMM) with T/S ratio as response variable, time of sampling (0.5 or 3 months) as a fixed effect, and individual chick ID as a random effect on the intercept. Next, we ran two further LMMs with the response variable T/S_0.5_ and T/S_3_, respectively to test the effect of parental ages on chick telomere lengths at either time point, and whether these effects differed with chick sex. For each of these two models we tested the fixed effects of the paternal and maternal age categories (either “young” or “old”, with “old” as the reference level). To test for sex-specific parental effects, we included chick sex as a fixed effect (with “male” as the reference level) and an interaction of chick sex with parental age in both the T/S_0.5_ and T/S_3_ models. As all chicks were sampled at 12 days post-hatching to collect T/S_0.5_ samples, but chicks were of a variable age when T/S_3_ samples were taken (mean = 100.8 days, s.d. = 8.4), we also included a fixed effect of the exact chick age in days, sample day, in the model with T/S_3_ as the response. We found no statistically significant effect of ‘sample day’ on T/S_3_ (posterior mode = 0.004, 95%CI (credible interval) = −0.02, 0.007, pMCMC = 0.46). Still, to account for any potential bias we retained “sample day” as a fixed effect in the T/S_3_ model. Note that our results, however, remained qualitatively similar whether or not we retained sample age in the model.

There is also a potential effect of the time a blood or DNA sample has been stored until analysis on quantified telomere lengths (Sibma, 2021). As the T/S_0.5_ samples were a mix of previously-, or newly-extracted DNA samples, we tested whether time of extraction had any effect on the calculated T/S_0.5_ ratio as a result of DNA degradation (Madisen et al., 1987) (*n* samples newly-extracted = 10/75). We fitted a LMM with T/S_0.5_ as the response and the time of extraction, sample age, as a fixed effect two-level categorical variable of, either “newly-“ or “previously extracted”. Newly-extracted samples were extracted at the same time as the 3 months samples, previously-extracted samples were extracted 18 months prior. We found no statistically significant difference between newly- and previously-extracted samples (posterior mode = −0.06, 95%CI = −0.20, 0.08, pMCMC = 0.389). Further, a previous study investigating house sparrow telomere length found that the repeatability between newly- and previously-extracted samples was moderate (0.45, 95%CI = 0.35, 0.63; Sibma, 2021).

We included the nest box ID and aviary ID in which chicks were born as random effects on the intercept in all models to account for variance between broods and aviaries. We also included the random term of qPCR plate ID in all models to account for between-plate variance on the intercept. We include a random effect of chick ID in the first model testing for a change in chick telomere length between the two time periods as we had multiple measurements from individuals. We examined the collinearity of the fixed effects, as collinearity could distort model results, in no cases did this exceed 0.7 (Dormann et al., 2013). All models were run using the Markov chain Monte Carlo (MCMC) method in the R package MCMCglmm v.2.29 (Hadfield, 2010).

### Model Validation

As we used a Bayesian modelling approach, we deemed fixed effects to be statistically significant if their 95% credible intervals did not span zero, we also report MCMC-p-values (pMCMC) for interpretation (Hadfield, 2010). All terms were retained in models irrespective of their statistical significance. We directly assessed model autocorrelation for fixed and random effects to ensure that the risk of type I errors was not inflated. We also inspected iteration and density plots to ensure that effects showed equal variation around a constant mode and demonstrated convergence (Gelman and Hill, 2006; Hadfield, 2010). We ran all models for 100,000 iterations with a thinning interval of 10 and used minimally-informative flat priors. Gelman-Ruben statistics for all variables was between 1 and 1.05 indicating convergence (Brooks and Gelman, 1998). Effective sample sizes were >400 at all times and trace plots indicated good mixing of chains. All statistical analyses were carried out in R v.3.6.1 (R Core Team, 2021).

## Results

**FIGURE 1:**
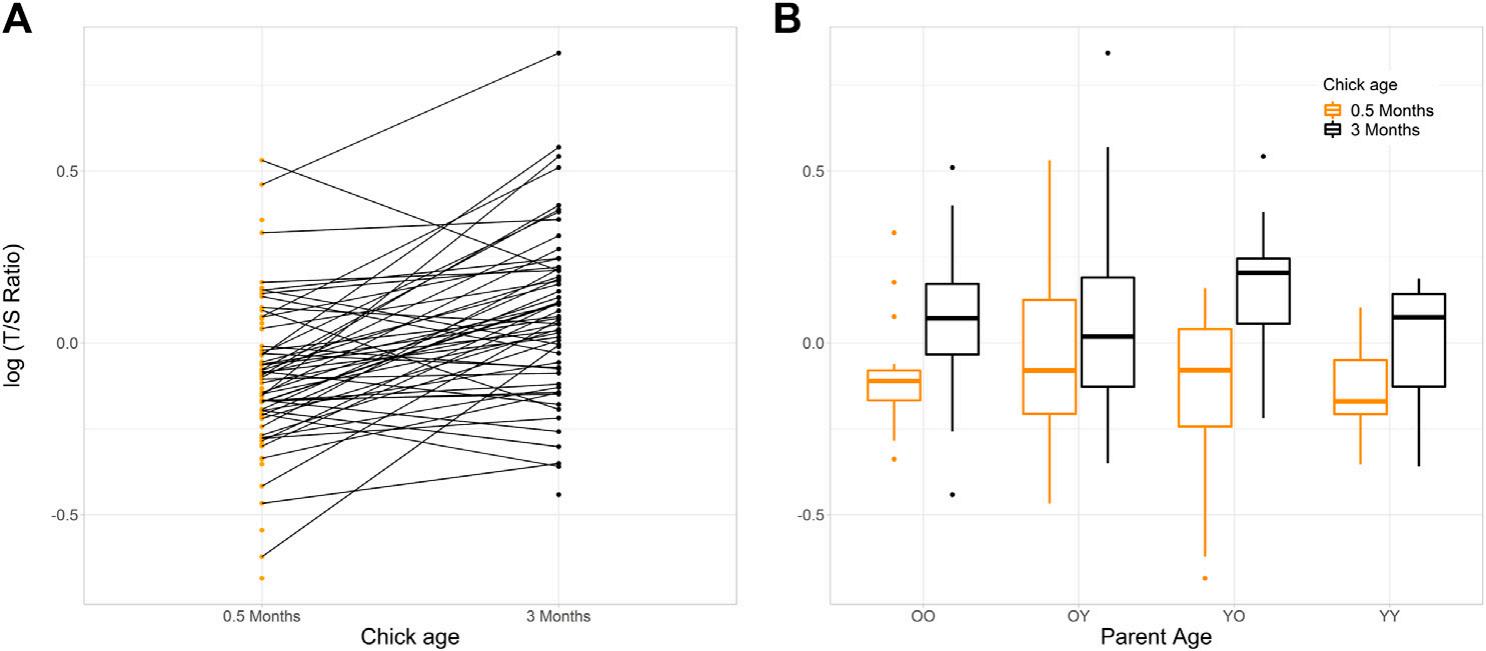
Change in telomere length log (T/S Ratio), within house sparrow chicks at 0.5 and 3 months of age. **(A)** Individuals are connected by a line (n offspring with samples at 0.5 months = 75, at 3 months = 59). **(B)** Boxplots show the mean (central line) and 25th and 75th percentiles (lower and upper box bounds respectively) of the log (T/S Ratio) within age group of the chicks’ parents (young birds, Y, were a parents that hatched the preceding summer, old birds, O, were parents that were ≥4 years old). T/S Ratio is presented on the log scale to aid visualisation. YO = young mothers, old fathers (*n* = 19 offspring with 0.5 months samples, 12 offspring with 3 months samples). OO = both parents old (*n* = 18, 19). OY = old mothers, young fathers (*n* = 17, 18). YY = both parents young (*n* = 15, 10).

Unexpectedly, the telomere length of 80.4% of the chicks for which both measurements were available increased between 0.5 and 3 months of age (*n* = 45/56; Figure 1 and Table 1).

**TABLE 1:** Results from a Bayesian MCMC linear mixed-effects model testing the difference between telomere length in house sparrow chicks at 0.5 and 3 months of age.

*Parameter*	*Estimate*	*95% credible interval*	*pMCMC*
***Intercept***	**0.93**	**0.84–0.99**	**<0.001**
***Chick age***	**0.19**	**0.12–0.26**	**<0.001**
***Random effects***			
*Chick ID*	0.00	0.00–0.01	
*Nest box ID*	0.02	0.01–0.04	
*Aviary ID*	0.00	0.00–0.00	
*qPCR plate ID*	0.00	0.00–0.01	
*Residual*	0.04	0.02–0.05	

Chick age was modelled as either 0.5 months (75 chicks) or 3 months (59 chicks), with 0.5 months as a reference level. Estimates shown are posterior modes. Statistically significant effects are shown in bold.

**FIGURE 2:**
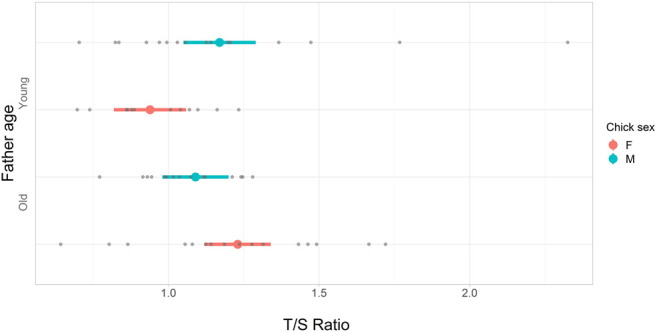
Forest plot of the posterior modes (red and blue dots) and corresponding 95% credible intervals (lines) from a linear mixed-effects model testing the relationship between T/S_3_, father age, and sex of chicks (Table 2). Fathers were assigned an age category of young, “Y”, or old, “O”. A young father hatched in the preceding summer, and an old father was >4 years old. Chick sex is indicated as either female, “red”, or male, “blue”. The number of offspring in each category; Y, and female = 12, male = 16, O, and female = 16, male = 15. Raw data points are shown as grey dots.

We did not find a statistically significant effect of parental age class on T/S at 0.5 months, which is shortly before sparrows gain independence and fledge from their nest (Table 2). However, we detected statistically significant effects of paternal age on T/S at 3 months such that daughters of young fathers had shorter telomeres than daughters of old fathers (Table 2; Figure 2). In contrast, paternal age had no statistically significant effect on the telomere length of sons at 3 months (Table 2; Figure 2).

**TABLE 2:** Results from two Bayesian MCMC linear mixed-effects models with telomere length of house sparrow chicks at age 0.5 months (*T/S_0.5_*) and 3 months (*T/S_3_*) as response variables, respectively.

*Parameter*	*T/S_0.5_*			*T/S_3_*		
	*Estimate*	*95% credible interval*	*pMCMC*	*Estimate*	*95% credible interval*	*pMCMC*
***Intercept***	**0.87**	**0.66–1.09**	**<0.001**	**1.57**	**0.32–2.71**	**0.022**
*Chick sex*	−0.13	−0.05–0.32	0.165	−0.23	−0.41–0.61	0.121
*Maternal age*	−0.08	−0.07–0.23	0.346	0.06	−0.32–0.21	0.711
*Maternal age x Chick sex*	0.06	−0.26–0.14	0.557	0.09	−0.21–0.43	0.573
***Paternal age***	−0.05	−0.26–0.14	0.557	−**0.27**	−**0.52**−**0.03**	**0.047**
***Paternal age x Chick sex***	−0.14	−0.34–0.07	0.177	−**0.40**	−**0.71**−**0.10**	**0.016**
*Sample age*	−0.01	−0.18–0.13	0.801	-	-	-
*Sample day*	-	-	-	0.00	−0.01–0.01	0.694
***Random effects***						
*Nest box ID*	0.01	0.00–0.03		0.01	0.00–0.02	
*Aviary ID*	0.00	0.00–0.01		0.01	0.00–0.01	
*qPCR plate ID*	0.00	0.00–0.01		0.00	0.00–0.01	
*Residual*	0.08	0.03–0.11		0.08	0.03–0.11	

Maternal and paternal age were modelled as either young or old; young birds hatched the preceding summer, and old birds were 4 years old). 0.5 months: *n* = 69 chicks, 3 months: *n* = 59. The reference level for parental ages was “old”, “female” for chick sex, and “old” for sample age. Estimates shown are posterior modes. Statistically significant effects are shown in bold.

## Discussion

Individual chick telomere length increased between 0.5 and 3 months of age. This increase disagrees with much of the published literature, which generally finds a decrease in telomere length in early life (De Meyer et al., 2007; Salomons et al., 2009; Boonekamp et al., 2014; Hoelzl et al., 2016; Cerchiara et al., 2017). While a population level increase in telomere length has previously been found in some long-lived bird species (Haussmann et al., 2007; Pauliny et al., 2012), other studies have found that telomere elongation for a proportion of chicks is more common in shorter-lived species (Eisenberg, 2019; Brown A. M et al., 2021; Brown T et al., 2021) and few longer-lived species (Cerchiara et al., 2017). For example, a study on jackdaws *Corvus monedula* found that between 5 and 30 days post-hatching, telomere lengths increased for 25% of sampled offspring (Grasman et al., 2011). An increase in early life telomere length has also been observed in non-avian taxa, including water pythons *Liasis fuscus* (Ujvari and Madsen, 2009) and European badgers *Meles meles* (Lieshout et al., 2019). Indeed, a recent paper by Heidinger et al. (2021) also found an increase in telomere lengths in some individuals in a population of house sparrows over time points years apart. A lack of comparable published research exploring a change in telomere length using multiple time points in early life may, in part, explain the surprising nature of our observed increase in telomere length in early life.

An increase in telomere length can have methodological (Sheldon et al., 2021) and/or biological explanations (Ujvari and Madsen, 2009). First, it could be due to DNA in samples degrading over time (Madisen et al., 1987; but see Seutin et al., 1991). Since we used previously-extracted DNA for the majority of 0.5 months samples, we investigated whether differential telomere degradation rates between DNA and blood sample extraction types could be a cause for the observed increase. We found no statistically significant difference between the telomere lengths of newly- and previously-extracted samples and thus, telomere degradation in extracted samples over time seems like an unlikely explanation for our results.

Second, qPCR plates contained both 0.5 and 3 months samples, and between-plate variance was negligible in all our models, highlighting that this element of our methodology had little impact on our results. Overall, we monitored procedural efficiency throughout data collection and did not identify any other potential methodological sources of variation; the repeatability estimates for the T/S ratios estimated from within-individual samples were well within the range of those for similar species in other qPCR studies (Kärkkäinen et al., 2021). Consequently, we believe that the increase in telomere length observed in our study has a biological explanation. For example, telomerase activity might have been maintained in the chicks after the first sample was taken. Indeed, two studies have shown that telomerase activity can be maintained up to 5 weeks post-hatching in zebra finches *Taeniopygia guttata* (Haussmann et al., 2007) and chickens *Gallus gallus* (Taylor and Delany, 2000). Yet, neither of these studies assessed telomerase activity at multiple time points in the same individual’s early life post-hatching, which remains an interesting future avenue for the field.

While we expected that old parents would produce chicks with shorter telomeres, as found in other short-lived bird species in line with predictions of the Lansing effect (Criscuolo et al., 2017; Bauch et al., 2019), our experimental approach found that old fathers produced daughters with longer telomeres, but only 3 months after hatching, potentially indicating an environmental or an age-dependent epigenetic effect (Matsushima et al., 2019). Positive relationships between parental age and offspring telomere length have previously been foundin long- and short-lived bird species (Asghar et al., 2015; Dupont et al., 2018; Brown A. M et al., 2021). Such effects may arise indirectly, for example, through improved parental care that older individuals provide compared to inexperienced, younger breeders. However, the positive effect of improved parental care by older parents also declines in the oldest individuals as they senesce (Beamonte-Barrientos et al., 2010; Becker et al., 2015). One potential cause for the lack of identifying an effect of maternal age on chick telomere lengths may result from a relative difference in the strength of maternal vs. paternal effects; if maternal effects are weaker, maternal effects may only be detectable with a larger sample size and as such may be present but undetectable in our study. Further, positive effects of parental age on telomere length are in constrast to some previous studies that instead found a negative effect of parental age resulting from the poorer condition of the oldest individuals (Criscuolo et al., 2017; Bouwhuis et al., 2018), or a lack of an effect of parental age on parental care (Nakagawa et al., 2007). In contrast, a recent study in house sparrows found no evidence for parental care effects and instead, stronger evidence for genetic effects of parental age on traits associated with telomere dynamics (Schroeder et al., 2015). It may be possible that the effects observed in our study arise from a phenomenon whereby parents that survive for longer are of an inherent higher genetic quality, and so produce higher quality offspring with longer telomere lengths relative to the average telomere lengths from offspring from younger parents which will include a wider range of adults of varying quality. Complicating this further is a study by Le Pepke et al. (2021) which found that environmental effects were the strongest predictors of telomere length in house sparrows. In addition, few studies investigating parental effects on telomere length continue to sample offspring telomere lengths into the post-fledging period, as in this study, and so will not be accounting for post-fledging parental care and how this may vary with parental age. Little is known about post-fledging parental care in house sparrows, however during this period juveniles tend to form flocks with their parents so it is likely that parental care will continue to be of some importance for chicks after they leave the nest, through e.g. continuing to provide food for young up to 14 days after they fledge (Summers-Smith, 1963). Positive effects of the ages of social parents have also been identified in house sparrows, which may indicate that the quality of individuals in an offspring’s social group post-fledging may also influence telomere lengths (Sibma, 2021). Evidently, future studies are required to further investigate post-fledging parental care for this species and others, and the potential effects of parental care in early life on offspring telomere dynamics.

While telomere lengths in offspring can be affected by an offspring’s environment (Dugdale and Richardson, 2018; Lieshout et al., 2021), effects of paternal age in birds have also been found to be independent of this (Boonekamp et al., 2014; Bauch et al., 2019). As such, there is overall growing support for at least contributory paternal, or z-linked, inheritance of telomere length in some species with a ZW sex-determination system (Olsson et al., 2011; Bouwhuis et al., 2018). These studies support our finding of positive effects of father age on daughters only. In further support of our findings is Schroeder et al. (2015) which identified a sex-specific heritable parental age effect on offspring fitness in house sparrows with daughters of older fathers having higher lifetime reproductive success than sons of older fathers. There is also increasing evidence demonstrating the potential positive effect of father age on offspring telomere lengths (sons and daughters: Brown A. M et al., 2021, daughters only: Dupont et al., 2018). A combined effect of z-linked inheritance and improved parental care or father quality for offspring of older fathers in our study may then explain why we find a positive effect of father age on daughter telomere lengths. Our results are then in contrast to studies finding that offspring telomere lengths correlate with mother age (Asghar et al., 2015; Reichert et al., 2015). However, effects of maternal age in these studies were identified at earlier time points than the ones used in this study (10 days in king penguins *Aptenodytes patagonicus*: Reichert et al., 2015; 9 days in great reed warblers *Acrocephalus arundinaceus*: Asghar et al., 2015). It may then be that while we found no effect of maternal age in our study, an influence of maternal age on chick telomere length may well have been present, but already diminished below detectable levels 0.5 months after hatching, when our first sampling took place.

We did not detect an effect of either maternal or paternal age on chick telomere length at 0.5 months after hatching. This is again in contrast to studies finding evidence to support the Lansing effect where offspring of older parents may have shorter telomeres in early life with potential implications for longevity (Priest et al., 2021b; Monaghan et al., 2020). Heidinger et al. (2016) similarly found no effect of parental age on offspring telomere length in early life at 25 days after hatching in European shags *Gulosus aristotelis*. Further, variation in pre-fledging telomere length may in part be explained by brood-specific additive genetic effects (Voillemot et al., 2012). As such, it may be that at later time points effects of parent age and post-fledging environmental factors appear to be more important than brood-specific effects in determining offspring telomere length. Again, there is a need for more studies investigating the relationship between paternal age and telomere dynamics to detect when and how patterns of telomere dynamics are driven.

In summary, we find that telomere lengths increased in early life with a likely biological cause. Furthermore, our results indicate that paternal age effects are more influential on offspring telomere length than maternal age effects in our population of house sparrows, with the daughters of older fathers having longer telomeres. Future analyses of telomerase activity levels in both the sperm of adult males would yield further insights into the drivers of parental age effects on offspring telomere dynamics in early life.

## Data Availability

The datasets presented in this study can be found in online repositories and associated R code. The names of the repository/repositories and accession number(s) can be found below: https://osf.io/6kwzh/.
